# Translation and psychometric validity of the Brazilian version of the European Organisation for Research and Treatment of Cancer—colorectal liver metastases module

**DOI:** 10.3332/ecancer.2022.1346

**Published:** 2022-01-17

**Authors:** Dabna Hellen Tomim, Bruna Eloise Lenhani, Luciana de Alcântara Nogueira, Tatiele Naiara Vogt, Leonel dos Santos Silva, Maria de Fátima Mantovani, Paulo Ricardo Bittencourt Guimarães, Luciana Puchalski Kalinke

**Affiliations:** 1 Department of Nursing, Federal University of Paraná, Av Prof Lothário Meissner 632, Curitiba, PR 80210-170, Paraná, Brazil; 2 Erasto Gaertner Hospital, R. Dr. Ovande do Amaral, 201, Curitiba, PR 81520-060, Paraná, Brazil; 3 High Risk Chemotherapy Service, Clinical Hospital of the Federal University of Paraná, R. Gen. Carneiro 181, Curitiba, PR, 80060-900, Paraná, Brazil; 4 Statistics Department, Federal University of Paraná, Av Cel Francisco H Santos 210, Curitiba, PR 81531-970, Brazil

**Keywords:** colorectal cancer, liver metastasis, EORTC, quality of life, validation

## Abstract

Patients with liver metastasis of colorectal cancer (CRC) experience important lifestyle changes that impair the quality of life (QoL). The use of instruments is important to assess the cancer patients’ QoL. To develop a Brazilian translation of the European Organisation for Research and Treatment of Cancer (EORTC) Quality of Life Questionnaire Colorectal Liver Metastases (QLQ-LMC21) questionnaire, and to investigate psychometric validity in patients with CRC with liver metastases, patients with liver metastasis from CRC answered the Brazilian versions of EORTC core Quality of Life Questionnaire-Core 30 (QLQ-C30) and the QLQ-LMC21 module, a demographic data questionnaire and a questionnaire to assess understanding against the translated QLQ-LMC21. Data collection was performed in three Southern hospitals in Brazil, from August 2017 to August 2019. Descriptive analyses and Spearman’s correlation tests were performed for construct and criterion validity. The Cronbach’s alpha test was performed to assess reliability. Significant values were *p* < 0.05. Ten patients participated in the study for the pilot test and 106 for validation, with a mean age of 58.2 + 12.4 years old. The translated questionnaire was easy to understand for the participants in the pilot test phase, with no need for modification. In the validation of the convergent construct, all the correlations were significant (*p* < 0.001) and with coefficients above 0.53. In the discriminant validity, among items of different scales, the values of the divergent correlations were pain scale (0.005 to 0.186) and fatigue (−0.01 to 0.186), all non-significant. In the criterion validation, the correlations were significant, *p* < 0.05, and higher among similar scales of the QLQ-LMC21 and QLQ-C30, *p*-value < 0.001. The total internal consistency of the questionnaire was adequate, with a Cronbach’s alpha value of 0.790. The Brazilian version of the QLQ LMC21 proved to be a valid reliable questionnaire to be used in conjunction with the QLQ-C30.

## Introduction

Colorectal cancer (CRC) is the third most frequent type of cancer worldwide, in both genders. In 2018, the estimate was more than 1.8 million new cases and 861,663 deaths worldwide due to CRC [1]. In Brazil, it is the second most frequent type of cancer. For each year of 2020 and 2021, the estimate is 20,470 new cases in women and 20,520 in men. In terms of mortality, in 2017, the number of deaths was 9,207 in men and 9,660 in women in Brazil [2].

In patients with CRC, mortality is high, mainly due to advanced metastatic disease. It is estimated that patients in stage IV, or metastatic CRC, has a 5-year survival rate of only 12% [3]. Liver metastasis is the most frequent in approximately 19%–31% of the patients with CRC, of whom 25% present metastasis at the time of diagnosis, and 45%–50% develop it after 2 years of resection of the primary tumour [4, 5].

The earlier the diagnosis and the performance of an adequate treatment, the greater the chances of survival of patients with CRC. Among the treatments available, surgical resection has been shown to be an effective method that provides increased survival or even a cure. However, many patients are not eligible for surgery due to the advanced stage of the disease, requiring palliative treatments to reduce symptoms and prolong survival [6].

Upon being diagnosed with CRC, the patient experiences important lifestyle changes, such as physical and emotional changes due to pain and discomfort, dependence and lack of self-esteem. The available treatments cause side effects, making the patients, in addition to having to endure emotional stress, also face physical difficulties arising from the therapy. Consequently, emotional problems such as depression can arise and, even if the treatment brings good results, the patients may experience limitations and feelings of anguish and distress, mainly due to the fear of relapse of the disease [7–9]. These factors impair the Quality of Life (QoL) of CRC patients. Therefore, knowing the symptoms and the effects of the therapy reported in the patient’s perception can assist in choosing the best treatment approach for each patient profile.

In the context of assessing the changes that CRC and the treatment cause in the QoL of the patients, research studies related to the theme are relevant, as they can measure the impact of the disease. For this purpose, the use of instruments that assess the QoL of cancer patients has been frequent. They have as their main objective to measure the real effects of the therapy in the patients’ lives. With the same objective, the European Organisation for Research and Treatment of Cancer (EORTC) developed questionnaires that evaluate the QoL of cancer patients in its various aspects. Among them is the Quality of Life Questionnaire-Core 30 (QLQ-C30), which has a function to assess physical, emotional and social issues, as well as symptoms and side effects of the treatment of cancer patients in general [10].

From the EORTC QLQ-C30 questionnaire, other complementary modules were developed to meet the particularities of different types of cancer. Questionnaires with the objective of assessing the QoL of CRC patients are among the most developed and used, possibly due to the position that CRC occupies in the estimates and to its impact on patients’ lives. Among the most used are the Functional Assessment of Cancer Therapy-Colorectal, developed by the American Functional Assessment of Chronic Illness Therapy (FACIT) and the Quality of Life Questionnaire Colorectal-29 (QLQ-CR29) developed by the EORTC [11, 12].

The FACIT has the Functional Assessment of Cancer Therapy-Hepatobiliary questionnaire, which measures the QoL of patients with hepatobiliary cancers, including metastatic CRC, hepatocellular carcinoma, pancreatic cancer and cancers of the gallbladder and bile duct [13].

Despite the impact on the health conditions that liver metastases due to CRC cause in the patients, specific questionnaires to assess QoL are scarce. Thus, the EORTOC Quality of Life Questionnaire Colorectal Liver Metastases (EORTC QLQ-LMC21) was developed with the purpose of measuring the QoL of patients with liver metastasis, originating from CRC [14, 15].

In Brazil, the EORTC QLQ-LMC21 instrument is not used and, so far, there is no knowledge of another instrument that has the same purpose. Therefore, this study had the purpose to develop a Brazilian translation of the EORTC QLQ-LMC21 questionnaire, and to investigate its psychometric validity in patients with liver metastases from CRC. The availability of the translated and valid questionnaire to be applied to the Brazilian population may help health professionals to better understand the patients’ needs and thus develop strategies aimed at better QoL.

## Methods

### Questionnaires

• Demographic and clinical form

To assess the characteristics of the study population, an instrument was applied to identify sociodemographic and clinical data. The variables identified were sex, marital status, age, occupation, education, location of the primary tumour, presence or absence of ostomy and chemotherapy protocol.

• Quality of Life Questionnaire-Core 30 (EORTC QLQ-C30)

The EORTC QLQ-C30 consists of 30 items that assess the individuals’ social, emotional and physical issues [10]. It consists of a global health scale, five items on the functional scale, three items on the symptom scale and six individual items on symptoms. It has been translated into more than 100 languages and its use is more frequent to measure QoL in clinical trials as patient-reported outcomes.

The answers to the questions in both questionnaires are on a Likert-type scale with four points corresponding to ‘no’ [1], ‘little’ [2], ‘moderately’ [3] and ‘a lot’ [4], except for questions 29 and 30 of the QLQ-C30, which vary on a scale from 1 to 7 points, with 1 corresponding to ‘terrible’ and 7 to ‘optimum’. The information on the scoring of the EORTC questionnaires is detailed in the QLQ-C30 scoring manual [16]. The QLQ-C30 is a valid instrument to be applied in Brazil, its psychometric properties of the Portuguese version are available in previous studies [17, 18].

• Quality of Life Questionnaire-Colorectal Liver Metastases (EORTC QLQ-LMC21)

The QLQ-LMC21 is a module of the QLQ-C30, which must be applied together. It aims to assess specific symptoms of patients with liver metastasis from CRC. The 21 items that make up the instrument are grouped into four scales (fatigue, nutrition, pain and emotional problems) and nine individual items (problems with taste, tingling in the hands, sore mouth, dry mouth, problems with jaundice and weight loss) [14, 15].

The EORTC Quality of Life department provided the QLQ-C30 questionnaires in Brazilian Portuguese and the original English version of the QLQ-LMC21 for translation and adaptation to Brazilian Portuguese and its scoring manuals, upon request of the authors of this study.

The values obtained for the QLQ-C30 and QLQ-LMC21 scores were transformed into scales from 0 to 100 according to the recommendations of the EORTC QoL manual [16]. The higher the values of the functional scales and of the global QoL, the better the patient’s QoL, while higher values in the symptom scales represent that the patient has more problems and symptoms, indicating worse QoL.

### Translation and adaptation

For the translation and adaptation of the QLQ-LMC21, the guidelines proposed by the translation manual provided by the EORTC [19] were followed. Two native Brazilian translators with a good command of the English language translated the English version of the questionnaire into Portuguese. The first had knowledge of the health field, and the second did not know the subject under study. The two versions translated into Portuguese were evaluated by a third professional translator and synthesized in a single version.

The Portuguese version of the QLQ-LMC21 was submitted for back-translation to two independent translators, with native English language and proficiency in Portuguese. In this process, the Portuguese version is again translated into English, so that possible errors can be identified during the translation process. Subsequently, all the translated and back-translated versions were forwarded to the EORTC translation team for evaluation, which availed the preliminary version of the QLQ-LMC21 in Portuguese for the pilot test.

The preliminary version of the questionnaire was applied to a sample of 10 patients with liver metastasis from CRC, who were undergoing chemotherapy treatment and who were native speakers of Brazilian Portuguese. At the end, the participants answered a questionnaire regarding its ease of comprehension. The results obtained from the pilot test were discussed among the researchers, and a report of this stage was forwarded to the EORTC for evaluation.

The final version of the QLQ-LMC21 in Brazilian Portuguese was defined and used in this study for the validation process, aiming to assess the validity and reliability in the study population.

### Patients

The participants eligible for this research were recruited from three chemotherapy outpatient clinics in hospitals located in the South region of Brazil. Collection was carried out in two different moments, the first for the pilot test of the QLQ-LMC21 that occurred between August and October 2017, and the second stage of validation between February 2018 and August 2019.

The participants had liver metastasis from CRC confirmed with imaging and/or histopathological examinations, were aged over 18 years old and received chemotherapy treatment. In the first phase of the research, there were 10 participants, as recommended by the EORTC [19]. In the second, 106 patients; the sample size for this second stage was defined following the reference of Pasquali [20], which should be five to ten participants per question.

### Interview procedure

The patients were approached and informed about the purposes of the study during chemotherapy sessions at the participating hospitals. They were interviewed only after accepting to participate in the study and signing the informed consent form. Each patient completed the Brazilian version of the QLQ-C30 core, the QLQ-LMC21 module and a demographic and clinical data questionnaire, once during chemotherapy treatment. All the questionnaires were applied by the same Nursing researcher, with experience in Oncology nursing, and a PhD student in the Nursing graduate programme.

In psychometric validation studies, it is important to consider the conditions and the environment in which the patients are approached for the application of the questionnaires. The less interference in the patients’ real usual environment, the more reliable their answers will be [21]. In this sense, in order to reduce research bias, in the three data collection hospitals, all the questionnaires were applied in similar environments and in the same situations: in the rooms where they usually receive treatment and during their chemotherapy sessions. This scenario is common for patients with hepatic CRC metastasis undergoing chemotherapy treatment in Brazil, which allows for the future application of the validated questionnaire to patients with similar characteristics and scenario, but in other study locations.

### Pilot testing

After defining the version translated into Brazilian Portuguese, the QLQ-LMC21 was applied to ten patients with liver metastasis from CRC for the pilot test stage. In this stage, the questionnaires were applied to collect sociodemographic and clinical data, namely: the QLQ-C30, the QLQ-LMC21 in Portuguese and a fourth instrument to identify the practicalities and difficulties encountered by the participants in relation to completing the translated questionnaire. If the patients reported any difficulties, they were asked if they had any suggestions for modifying the question. The EORTC Quality of Life team evaluated the report produced by the result of the pilot test.

### Ethics

The study was approved by the Committees of Ethics and Research with Human Beings of the Clinical Hospital of the Federal University of Paraná (Informed Consent Number: 2.137.221), of the Erasto Gaertner Hospital (Informed Consent Number: 2.822.368) and of the Ministro Costa Cavalcanti Hospital (Informed Consent Number: 2.592.656). The research was developed following the rules of the Resolution 466/12 of the National Health Council for research with human beings [22].

### Statistical analysis

All the data were analysed using the Statistical Package for Social Sciences software, version 20.0.

The characterisation data of the participants according to sociodemographic and clinical variables, both from the pilot test stage and from the participants in the validation stage, were analysed using descriptive statistics and presented in means and frequency.

The values of the descriptive analysis of the scores of QLQ-C30 and QLQ-LMC21 were converted into a scale from 0 to 100, and summarised in mean, minimum, maximum and standard deviation (SD). The results were considered significant for *p*-values below 0.05.

To assess the construct validity of the EORTC QLQ-LMC21 questionnaire, the Spearman’s non-parametric correlation coefficient was calculated. Convergent validity asserts that tests that evaluate the same or similar construct are strongly correlated. One of the methods applied to test convergent validity is to correlate the scores between two assessment tools or tools’ sub-domains that are considered to measure the same construct [23].

In this study, the convergent validity was tested by means of the correlations of the items that are constituents of the same subscale. For example, the evaluation of the correlation between the score of item ‘31. Have you had trouble with eating?’ and the total score of its respective ‘nutritional problems’ subscale.

Evidence for discriminant validity is provided when measures of constructs that theoretically should not be highly related to each other are, in fact, not found to be related to each other [24]. In this study, to assess the divergent validity, the correlations between the items of different subscales were analysed, that is, the correlation between items constituting subscales that have the function of measuring different domains. For example, the correlation of the score of item ‘31. Have you had trouble with eating?’ (which is part of the ‘nutritional problems’ scale) with the total score of the ‘fatigue’ subscale.

In this sense, the hypothesis is that the correlations of convergent validity must be statistically stronger than the correlations of divergent validity. The convergent and discriminant validity was analysed with the Spearman’s correlation coefficient, with a *p*-value < 0.05 indicates a significant association.

To assess criterion validity, the similar subscales of the EORTC QLQ-C30 and the EORTC QLQ-LMC21 questionnaires were analysed with the Spearman’s correlation coefficient. Criterion validation is performed by comparing a new scale with an already known one called ‘gold standard’; however, as there is no other scale that evaluates the QoL of patients with liver metastasis from CRC, in this study it was decided to perform the comparison of the EORTC QLQ-LMC21 questionnaire with the EORTC QLQ-C30, whose domains are similar. The scores of the ‘nutritional problems’ subscales of QLQ-LMC21 were correlated with the ‘nausea and vomiting’ subscale of QLQ-C30, ‘fatigue’ subscale of each questionnaire, ‘pain’ subscale of each questionnaire and ‘emotional problems’ of QLQ-LMC21 and ‘emotional performance’ of QLQ-C30. The hypothesis is that the correlation between similar subscales for each questionnaire is greater than the comparison between subscales that measure different domains between QLQ-C30 and QLQ-LMC21.

The reliability of the QLQ-LMC21 was assessed using the Cronbach’s alpha coefficient to access the internal consistency of the instrument, moderate values being determined from 0.41 to 0.60; substantial, from 0.61 to 0.80; and almost perfect, from 0.81 to 1 [25].

## Results

The final sample for the validation phase of this study was 106 participants, with liver metastasis from CRC, coming from three hospitals that provide cancer treatment. The questionnaire compliance of the 106 patients was 100% in this study. The only missing data was for one subject who omitted the answer of the question about sexual function. The sample consisted of 63 men (59.43%) and 43 women (40.57%), with an age range between 32 and 86 years old and a mean of 58.2 years old (SD = 12.4 years old). They were predominantly married (60.38%, *n* = 64) with two to three children (55.56%, *n* = 59). Regarding schooling, 45 of the patients (42.45%) had less than 8 years of study and 9 (8.49%) had some higher education. As for occupation, 38 (35.85%) participants reported that they were receiving some benefit due to the impossibility of working for health reasons, and 30 (28.30%) were receiving retirement payments due to length of service or age.

In this study, 45 (42.45%) patients had an ostomy. The predominant chemotherapy regimen, 55 (51.89%), was the combination of 5-Fluorouracil and Leucovorin with Oxaliplatin. The colon region was responsible for 41 (38.68%) of the primary tumours, and the rectum for 33 (31.13%).

### Descriptive analysis of QLQ-C30 and EORTC QLQ-LMC21

Table 1 shows the descriptive measures of all the domains of the QLQ-C30 and QLQ-LMC21 scale. In relation to the QLQ-C30, the physical performance (*M* = 81.07, SD = 18.95) and cognitive performance (*M* = 83.49, SD = 24.93) items presented better scores in relation to function (*M* = 75.47, SD = 31.47), social (*M* = 77.36, SD = 28.10) and emotional (*M* = 74.45, SD = 26.98), the latter being the most affected domain, since it has the lowest score in relation to the other items. When analysing the symptom scale, the most affected domains were in relation to financial issues (*M* = 33.65, SD = 38.63), followed by insomnia (*M* = 25.16, SD = 38.71), fatigue (*M* = 24.11, SD = 25.03) and pain (*M* = 22.96, SD = 29.77). The least affected symptom was dyspnoea (*M* = 5.66, SD = 16.89) (Table 1).

In relation to the data obtained from the QLQ-LMC21, the most expressive domains were emotional problems (*M* = 43.08, SD = 26.36) and fatigue (*M* = 40.15, SD = 27.50). Regarding the individual items, sex life (*M* = 45.08, SD = 45.29), peripheral neuropathy (*M* = 37.74, SD = 38.23) and dry mouth (*M* = 36.48, SD = 38.63) were the most affected (Table 1).

### Convergent and divergent construct validation

In this stage, analyses of the correlations between items of the same subscale and items of different subscales of the QLQ-LMC21 were performed. The correlation coefficient between items of the same subscale was used to assess convergent validity. All the correlations were significant (*p* < 0.001) and with coefficients above 0.53, with emphasis on the correlations between items on the nutritional problems subscale, which ranged from 0.75 to 0.80, and items on the fatigue subscale, which ranged from 0.78 to 0.79. For the divergent validity, the correlation coefficient was evaluated between items of different subscales of the QLQ-LMC21, whose most divergent values were items from the pain (−0.005 to 0.186) and fatigue (−0.01 to 0.186) subscales when compared to items from other subscales of the QLQ-LMC21. It was possible to observe that the correlations between items of the same subscale were higher when compared to different subscales of the QLQ-LMC21, demonstrating good construct validation (Table 2).

### Concurrent criterion validation

To assess the validation of concurrent criteria, a correlation was performed between the EORTC QLQ-C30 scales and the EORTC QLQ-LMC21 module, with data presented in Table 3. All the correlations were significant; however, they are not considered high correlations. It is highlighted that the highest correlation values were between similar scales of the QLQ-LMC21 and QLQ-C30 (pain × pain = 0.45; fatigue × fatigue = 0.49; nausea and vomiting × nutritional problems = 0.45). Therefore, the QLQ-LMC21 questionnaire has similarities when comparing scales that measure constructs similar to the QLQ-C30, and is capable of assessing specific characteristics that are not covered by the QLQ-C30, demonstrating that the instrument has criterion validity, since it is able to evaluate what is intended to.

### Reliability

The reliability of the QLQ-LMC21 was assessed by its internal consistency, with Cronbach’s alpha coefficient calculation. In this analysis, the overall internal consistency of the instrument was assessed first, with a Cronbach’s alpha value of 0.790. The scale that showed the lowest internal consistency was that of nutritional problems, with an index of 0.541, which is still considered moderate consistency. The index of fatigue scale was 0.709, pain scale was 0.605 and emotional problems was 0.632.

When assessing the internal consistency by excluding each item from the questionnaire (Table 4), it was possible to observe that the Cronbach’s alpha increases to 0.801 with the exclusion of the ‘Have you had numbness (tingling) in your hands or feet?’ item. In addition, it rises to 0.791 when excluding the ‘Did your disease or treatment affect your sex life (for the worse)?’ item. These results demonstrate that the exclusion of any item does not significantly alter the general internal consistency of the questionnaire. Thus, there was no need to modify or exclude any item from the questionnaire.

## Discussion

This study aimed to translate and evaluate the validity and reliability of the QLQ-LMC21 module of the EORTC questionnaire in the Portuguese version for a sample of Brazilian patients with liver metastasis from CRC. Our results demonstrated that the QLQ-LMC21 translated into Brazilian Portuguese was easy to understand by the patients, with good psychometric validity, with convergent and divergent construct validity, criterion validity and good internal consistency.

For this study, the QLQ-LMC21 was used in addition to the QLQ-C30. All the translation stages were performed in agreement with the EORTC team. The preliminary version translated into Portuguese was applied to a sample of ten participants for the pilot test, who reported having no difficulty in completing the translated questionnaire and did not suggest any modification of terms or words. These results demonstrate that the translated version of the QLQ-LMC21 into Brazilian Portuguese was well accepted and considered understandable for its use by the patients.

There are few studies published in the literature that validated QLQ-LMC21; therefore, our results were compared to those found in the validation of the original and the Polish versions of QLQ-LMC21. To assess validity and reliability, the QLQ-LMC21 module translated into Portuguese was applied to a sample of 106 Brazilian patients with liver metastasis from CRC.

Therefore, questionnaires that assess QoL are considered essential tools for the area of Oncology. They measure QoL by the patient’s own perception. This assessment is important in clinical trial research studies to define which treatment can be the most appropriate for the patient’s profile, through a patient-reported outcome. In multicentric surveys, they allow for comparisons between populations of different cultures. In addition, questionnaires of this nature can provide pertinent information about the patient’s real situation and provide a survival forecast based on their answers [26].

In Brazil, there was no questionnaire to measure the QoL of patients with liver metastasis from CRC. In this scenario, the study of the translation and validation of the QLQ-LMC21 made it possible to apply it together with the QLQ-C30 to a sample of Brazilian patients. Based on the results obtained with the QLQ-C30, it was possible to demonstrate that cognitive and physical functioning had better scores in relation to roles, social and emotional functioning, the latter being the most affected domain due to its lower score in relation to the other items. These data are similar to the validation study of the original QLQ-LMC21 and of the Polish version, in which the domains with the best scores were cognitive function and physical function. However, unlike this Brazilian study, emotional function had better scores in relation to social function, that is, the social domain was the most impaired in the assessment of QoL in these studies [14, 27].

In this Brazilian study, it was possible to observe that the domains with the most affected scores were emotional problems and fatigue. In relation to the individual items, the most affected one was sex life. These data are similar to the validation study of the original instrument and to the validation of the Polish version, in which the most affected domains were emotional problems and fatigue and, of the individual items, the most affected domain was sex activity [14, 27].

Construct validation was performed by assessing correlations between items of the same subscale and items of different subscales of the QLQ-LMC21. In this study, we confirmed the hypothesis that the translated version of the QLQ-LMC21 showed good construct validity, since all the correlations between items of the same subscale of the QLQ-LMC21 were significant and with values above 0.5, while the correlations between items of different subscales obtained lower and non-significant values. These results are similar to the validation of the Polish version of the QLQ-LMC21. Correlation values above 0.40 were assumed, and it was found that all the item correlations were significantly higher when compared to their own subscale than with other subscales [27].

Other translation and validation studies of EORTC questionnaires also used this type of test to assess construct validation, with correlation of items in the subscale with the different subscales belonging to the same questionnaire. Among them are: the translation and validation study of the Quality of Life Questionnaire Brain-20 (QLQ-BN20), which assesses the QoL of brain cancer patients, for use in Iran [28]; the Quality of Life Questionnaire Core 15 for Palliative Care, to assess palliative care QoL, for use in Croatia [29]; and QLQ-CR29, to assess the QoL of patients with CRC, for the Chinese version of Taiwan [30]. All performed convergent and divergent validation, and there was a greater correlation between comparisons of items in the same subscale than in different subscales.

The criterion validation in the present study was of the concurrent type, with the correlation between the QLQ-C30 scales and the QLQ-LMC21 scales. As expected, the correlations between the scales that measure different dimensions were weak, except for the comparison between the pain, fatigue and nausea and vomiting scales with nutritional problems in both questionnaires, which showed higher correlation values. These results are similar to the studies that evaluated criterion validity by correlating the QLQ-LMC21 with the QLQ-C30, which found weak correlations between the questionnaires’ scales, except for the comparison between similar scales of both instruments [14, 27].

Other validation studies of EORTC questionnaires, for other languages, carried out the correlation tests of their scale with the scales of QLQ-C30, possibly due to the non-existence of another instrument that assessed the same criterion with confidence. As an example, the validation of the Chinese versions of QLQ-BN20 [31] and of QLQ-CR29 [32], which correlated the items of their scales with the items of QLQ-C30 and found higher correlations between items of similar scales, corroborating with the present study.

As for reliability, this study demonstrated that the version of the QLQ-LMC21 translated into Brazilian Portuguese showed good internal consistency by means of the analysis of the Cronbach’s alpha index. The overall value of the instrument was 0.790, considered substantial [25].

When the reliability values for each QLQ-LMC21 scale is analysed, it was possible to observe that the one with the lowest values was the scale of nutritional problems. These results are partly similar to the validation study of the Polish version. When assessing patient reliability before treatment for hepatectomy, the scale with the lowest reliability value was the scale of nutritional problems. On the other hand, in this same study, when patient reliability at follow-up, palliative or hepatectomy was analysed, the lowest reliability value was for the pain scale. This result may have been different in our study due to the smaller sample of patients. Another possible cause for this result is education, which, unlike the study by Polish version that most participants had a technical level, in our study most patients had less than 8 years of study which could cause a greater variability in the responses of the items nutritional problems, decreasing the value of reliability [27].

With item exclusion analysis, what would provide the highest index is the item related to numbness (tingling) in the hands or feet. However, the increase in the Cronbach’s alpha index would be from 0.790 to 0.801, not considered significant, which demonstrates that there is no need to exclude or modify any items of the scales.

The antineoplastic chemotherapy treatment most used by the participants of this study was with Oxaliplatin. It has some side effects, including peripheral neuropathy, which can cause greater sensitivity or pain aggravated by cold [33]. This fact highlights the importance of assessing the effects that the treatment can have on patients with CRC liver metastasis, which demonstrates the importance of not excluding this question in the questionnaire due to the insignificant increase in the Cronbach’s alpha index.

There are some limitations in the study: due to the specificity of the characteristics of the target population, it was not possible to carry out other types of validation, such as clinical validation by means of known groups. The difficulty in obtaining a sufficient number of participants made it impossible to assess separate groups that would receive only palliative treatment, or hepatectomy, or to perform two measurements before and after the treatment of choice.

## Conclusion

The translated version of the QLQ-LMC21 into Portuguese applied to a sample of Brazilian patients with CRC liver metastasis was found to be reliable and valid, with good results measured by the instrument’s internal consistency, construct validity and criterion validity. The QLQ-LMC21 is then appropriate to measure the QoL of Brazilian patients with liver metastasis from CRC.

The availability of a valid and reliable questionnaire helps the professionals when evaluating new treatment options, identifying which domains of the QoL of these patients are most affected and thus elaborating targeted goals that provide a better QoL to this population.

## Conflicts of interest

The author(s) declare that they have no conflicts of interest.

## Funding statement

This study received financial assistance through a doctoral scholarship provided by the Coordination for the Improvement of Higher Level Personnel (Coordenação de Aperfeiçoamento de Pessoal de Nível Superior, CAPES - 88881.311846/2017-01).

## Authors’ contributions

Study conception and design: DHT, LPK; data collection: DHT, BEL, LSS; data analysis and interpretation: DHT, PRBG, LPK, TNV; drafting of the article: DHT, LPK, MFM, LAN; critical revision of the article: DHT, LPK, PRBG.

## Figures and Tables

**Table 1.** Descriptive measures of the QLQ-C30 and QLQ-LMC21 domains (*n* = 106).

Variables	*n*	Mean	SD	Minimum	Maximum
**QLQ-C30**					
Global QoL	106	75.08	19.33	25	100
**Functional scale**					
Physical performance	106	81.07	18.95	26.67	100
Function performance	106	75.47	31.47	0	100
Emotional performance	106	74.45	26.98	0	100
Cognitive performance	106	83.49	24.93	0	100
Social performance	106	77.36	28.10	0	100
**Scale of symptoms**					
Fatigue	106	24.11	25.03	0	100
Nausea and vomits	106	8.33	16.63	0	100
Pain	106	22.96	29.77	0	100
Dyspnoea	106	5.66	16.89	0	100
Insomnia	106	25.16	38.71	0	100
Loss of appetite	106	19.81	34.97	0	100
Constipation	106	17.30	32.28	0	100
Diarrhoea	106	15.72	30.59	0	100
Financial difficulties	106	33.65	38.63	0	100
**QLQ-LMC21**					
**Scales**					
Nutritional problems	106	18.24	25.57	0	100
Fatigue	106	40.15	27.50	0	100￼
Pain	106	18.66	22.57	0	100
Emotional problems	106	43.08	26.36	0	100
**Individual items**					
Weight loss	106	23.27	37.42	0	100
Taste	106	29.25	38.96	0	100
Dry mouth	106	36.48	38.63	0	100
Sore mouth	106	13.21	28.98	0	100
Peripheral neuropathy	106	37.74	38.23	0	100
Jaundice	106	6.92	21.44	0	100
Contact with friends	106	13.84	29.41	0	100
Talking about feelings	106	10.69	27.81	0	100
Sex life	105^a^	45.08	45.29	0	100

Note: reference: SD, Standard deviation

a Missing data

**Table 2.** Spearman’s correlation coefficient between items of the same subscale and with items of different subscales of the QLQ-LMC21 (n = 106).

Correlation amplitude of the coefficients	Correlation of the items belonging to the same subscale __________________ Convergent validity	*p*-values	Correlation of the items belonging to different subscales _____________________ Divergent validity	*p*-values
Nutritional problems	0.75–0.80	0.001^a^–0.001^a^	0.04–0.17	0.647–0.087
Fatigue	0.78–0.79	0.001^a^–0.001^a^	−0.01–0.186	0.936–0.056
Pain	0.64–0.79	0.001^a^–0.001^a^	−0.005–0.186	0.956–0.056
Emotional problems	0.53–0.83	0.001^a^–0.001^a^	0.056–0.131	0.567–0.181

Note:

a *p* < 0.001

**Table 3.** Spearman’s correlation coefficient between the QLQ-C30 and QLQ-LMC21 scales (*n* = 106).

QLQ-LMC21	Nutritional problems	*p*	Fatigue	*p*	Pain	*p*	Emotional problems	*p*
QLQ-C30								
Physical performance	−0.33	0.000	−0.46	0.000	−0.49	0.000	−0.31	0.001
Function performance	−0.27	0.005	−0.47	0.000	−0.43	0.000	−0.48	0.000
Emotional performance	−0.27	0.005	−0.27	0.005	−0.31	0.001	−0.58	0.000
Cognitive performance	−0.19	0.051	−0.34	0.000	−0.32	0.001	−0.23	0.018

**Table 4.** Internal consistency for each item excluded from QLQ-LMC21.

Items	Cronbach
31. Have you had trouble with eating?	0.785
32. Have you felt full up too quickly after beginning to eat?	0.782
33. Have you worried about losing weight?	0.790
34. Have you had problems with your sense of taste?	0.784
35. Have you had a dry mouth?	0.787
36. Have you had a sore mouth or tongue?	0.789
37. Have you been less active than you would like to be?	0.770
38. Have you had tingling hands or feet?	0.801
39. Have you had pain in your stomach area?	0.781
40. Have you had discomfort in your stomach area?	0.784
41. Have your skin or eyes been yellow (jaundiced)?	0.790
42. Have you had pain in your back?	0.777
43. Have you felt slowed down?	0.778
44. Have you felt lacking in energy?	0.777
45. Have you had trouble having social contact with friends?	0.783
46. Have you had trouble talking about your feelings to your family or friends?	0.785
47. Have you felt stressed?	0.785
48. Have you felt less able to enjoy yourself?	0.778
49. Have you worried about your health in the future?	0.772
50. Were you worried about your family in the future?	0.775
51. Has the disease or treatment affected your sex life (for the worse)?	0.791

Source: Specimen from EORTC (2020).

^a^The analyses were made with the Portuguese version from EORTC (2020).
